# The effects of virtual reality interventions on outcomes related to well-being among autistic children: a systematic review and meta-analysis of randomized controlled trials

**DOI:** 10.3389/fpsyg.2026.1924814

**Published:** 2026-09-23

**Authors:** Yangkai Zhang, Chuanxia Chen, Wenhu Zheng, Shiyan Luo

**Affiliations:** 1 Basic Education Research Center, Faculty of Education, Southwest University, Chongqing, China; 2 Chongqing No. 2 Foreign Language School, Chongqing, China; 3 Center for Studies of Education and Psychology of Ethnic Minorities in Southwest China of Southwest University, Chongqing, China

**Keywords:** autism spectrum disorders, children, meta-analysis, virtual reality, well-being

## Abstract

**Background:**

Virtual reality (VR) interventions have been increasingly employed in therapeutic and educational contexts for autistic children.

**Objective:**

To conduct a systematic review and meta-analysis of randomized controlled trials to examine the effects of VR interventions on outcomes related to subjective well-being (SWB) and psychological well-being (PWB) among autistic children.

**Method:**

This meta-analysis was performed in accordance with the PRISMA guidelines. First, seven electronic databases were searched: PubMed, Embase, Cochrane Library, Web of Science, PsycINFO, ProQuest Central, and IEEE Xplore. Subsequently, ten eligible studies were identified. Finally, data extraction was conducted, and effect sizes were pooled using random-effects models for SWB-related outcomes and PWB-related outcomes, respectively. Heterogeneity tests, sensitivity analyses, publication bias tests, and quality assessment were also performed.

**Results:**

VR yielded a positive yet nonsignificant effect on SWB-related outcomes (g = 0.37, 95% CI [−0.03, 0.78], z = 1.80, *p* = 0.07), whereas VR produced a statistically significant positive effect on PWB-related outcomes (g = 0.45, 95% CI [0.27, 0.63], z = 4.87, *p* < 0.05).

**Conclusion:**

Pooled analyses revealed a statistically significant positive effect of VR interventions on PWB-related outcomes among autistic children. However, this finding should be interpreted with caution given that 70% of included randomized trials had some concerns regarding risk of bias and only eight effect sizes contributed to this pooled estimate. Current evidence supports tentative promise for VR within research-oriented clinical and educational contexts rather than immediate real-world clinical application, while its potential for enhancing SWB-related outcomes remains to be further investigated.

**Systematic review registration:**

https://www.crd.york.ac.uk/PROSPERO/view/CRD420261411309

## 1 Introduction

Autism spectrum disorder (ASD), a heterogeneous neurodevelopmental disorder, is characterized by deficits in social communication, restricted interests, and repetitive behaviors (First, 2013). The precise etiology of ASD remains elusive. However, existing studies have indicated that a combination of genetic, environmental, and neurodevelopmental factors may contribute to its development (Loomes et al., 2017). The reported prevalence of ASD has increased over the past decade. Data from the Autism and Developmental Disabilities Monitoring (ADDM) indicated a prevalence of 1 in 60 in 2014, 1 in 54 in 2016, and, for 2020, 1 in 34 among boys compared with 1 in 145 among girls (Knopf, 2020).

Autistic children, as well as their parents, are facing immense difficulties. Autistic children encounter multiple challenges in social and nonverbal communication (Billstedt et al., 2005), facial emotion processing (Loth et al., 2018), and social interactions (Knott et al., 2006). Such challenges often lead to increased social isolation and reduced quality of life for autistic children (Hirota and King, 2023). Parents of autistic children face an elevated risk of chronic stress (Hayes and Watson, 2013; Miodrag and Hodapp, 2010). Factors such as concerns about their children’s intellectual functioning and mastery of adaptive behaviors, worries about parent–child relationships, their children’s high risk of exposure to adverse childhood experiences, and uncertainty about their children’s futures can contribute to anxiety and distress (Bermúdez et al., 2015; Rivard et al., 2014; Hutchison et al., 2016). In addition to these emotional challenges, parents of autistic children also face a number of financial problems. In the United States, the costs associated with caring for autistic children are about four times greater for medical expenditures and about 8–9 times greater for general expenditures compared to the costs of caring for non-autistic children (Ganz, 2007).

The aforementioned array of difficulties stemming from ASD renders it imperative to develop effective intervention strategies for autistic children. A range of evidence-based intervention strategies have been developed for autistic children, including behavioral therapy, developmental therapy, sensory integration therapy, social skills training, pharmacological treatments, psychological therapies, and other alternative intervention methods (Qin et al., 2024; Hirota and King, 2023; Hyman et al., 2020). In clinical practice, a comprehensive treatment approach, which includes pharmacological interventions, behavioral correction, educational training, and physical therapy, is commonly employed.

Research has indicated that traditional interventions (as mentioned above) for autistic children may be inaccessible, costly to implement, and ineffective due to insufficient available resources and low participant motivation (Shane and Albert, 2008).

However, Industry 4.0 technological innovations have opened new avenues for the screening and intervention of ASD. Virtual reality interventions have gained increasing attention in ASD rehabilitation. Virtual reality (VR) refers to a three-dimensional digital representation of real or imagined social and behavioral contexts, which enables users to interact with them (Howard et al., 2021). VR systems vary substantially in level of immersion, spanning non-immersive desktop platforms to immersive systems including head-mounted displays (HMDs) and cave automatic virtual environment (CAVE) systems (Munson and Pasqual, 2012). In immersive virtual reality systems, users are embedded within computer-generated virtual worlds (Wallace et al., 2010; Lorenzo et al., 2016), with their visual perspective dynamically updated in accordance with physical positional changes. Such systems deliver enhanced environmental realism and permit more interactive user engagement. In contrast, desktop virtual reality environments offer restricted interactive capabilities, as all visual stimuli and user experiences are mediated through a conventional computer screen.

Empirical studies have verified that VR interventions benefit autistic children in multiple domains. They improve social skills (Kouhbanani et al., 2021; Pv et al., 2023; Mittal et al., 2024), postural balance (Falivene et al., 2025; Abdel Ghafar et al., 2025), self-care abilities and emotion regulation (Ma and Song, 2025), communication and problem-solving skills (Turnacioglu et al., 2022; Herrero and Lorenzo, 2020), mental health (Lunsky et al., 2025), parasympathetic nervous function (Rodrigues et al., 2025), and job interview proficiency (Smith et al., 2021). Evidence also indicates that VR interventions can mitigate specific phobias and fears (Maskey et al., 2014; Maskey et al., 2019) as well as relieve anxiety symptoms (Simões et al., 2018; Genova et al., 2021) among autistic children.

Meta-analysis, a robust and rigorous research method, can yield evidence of high quality to guide clinical practice and facilitate subsequent academic exploration. Previous meta-analyses have synthesized evidence regarding VR interventions for autistic children. Most of these reviews have focused on effect sizes of outcome indicators, including social skills, emotion recognition (Herrero and Lorenzo, 2020; Li et al., 2023; Mittal et al., 2024; Yang et al., 2025; Cui et al., 2026; Mesa-Gresa et al., 2018; Farashi et al., 2024), communication skills (Wang et al., 2026), core autistic deficits and functional performance (Sun et al., 2026), motor ability (Guan et al., 2026), and daily living competence (Karami et al., 2021). To date, although several prior meta-analyses have indirectly covered partial well-being-related outcomes (e.g., Mittal et al., 2024; Yang et al., 2025; Cui et al., 2026), none have separately pooled effect sizes categorized by distinct subtypes of well-being. However, well-being plays a crucial role in facilitating the healthy physical, mental, and social development of children, and higher levels of well-being may help them build resilience and adapt more effectively to daily challenges. Therefore, focusing on autistic children, the present study examines the intervention effects of virtual reality on well-being-related outcomes to answer the following research questions:

1. What are the intervention effects of virtual reality on well-being-related outcomes among autistic children?
2. Are there differences in the intervention effects of virtual reality on different types of well-being-related outcomes?

## 2 Methods

The present systematic review and meta-analysis was conducted in accordance with the PRISMA 2020 guidelines (Page et al., 2021). This systematic review and meta-analysis was prospectively registered on PROSPERO (Registration ID: CRD420261411309).

### 2.1 Inclusion and exclusion criteria

Studies that met all of the following criteria were included in this review:

1. Participants: Participants were autistic children (aged ≤ 19 years).
2. Interventions: VR must be the core intervention component. For combined intervention protocols, the independent effect of VR should be extractable for separate analysis.
3. Controls: Eligible control groups were established without VR intervention, including placebo, treatment as usual, education, and attention placebo control.
4. Outcome measures: Studies were required to report complete well-being-related outcomes, as well as full quantitative data (means, standard deviations, sample sizes, or effect sizes) for meta-analytic pooling.
5. Study design: All included studies were RCTs, including individually randomized RCTs and cluster RCTs, with standardized designs and complete procedures for interventions and controls.
6. Others: Published in English with accessible complete data.

Studies that met any of the following criteria were excluded:

1. Participants: Studies targeting non-autistic children; the age of participants was older than 19 years; studies with mixed clinical samples where independent data of ASD subgroups could not be extracted.
2. Interventions: Studies without VR as an intervention component, or combined interventions where VR merely acts as an auxiliary element with non-extractable independent effects.
3. Controls: Studies adopting ineligible control regimens, such as other novel interventions and medication control.
4. Outcome measures: Studies that reported no well-being-related indicators; studies that only described well-being-related outcomes without complete or available quantitative data for meta-analysis.
5. Study design: Non-randomized studies, including observational studies (cross-sectional, cohort, and case–control studies), uncontrolled quasi-experiments, case reports, case series, reviews, systematic reviews, meta-analyses, guidelines, editorials, commentaries, and other grey literature.
6. Others: Non-English publications; duplicate publications (only the latest and most complete version was retained); conference abstracts, brief reports, and ongoing studies without full texts and complete data; sub-studies and derivative studies with highly overlapping samples with already included studies.

Although VR immersion levels constitute an important source of heterogeneity across included studies, the primary research question of this meta-analysis aimed to examine the overall effect of VR-related interventions, with a focus on the impact of VR usage on well-being among autistic children. Specifically, the comparison was conducted between the experimental group receiving VR interventions and the control group without VR exposure. Accordingly, immersion level was not considered in the inclusion and exclusion criteria.

### 2.2 Search strategy

A comprehensive literature search was conducted across PubMed, Embase, Cochrane Library, Web of Science, PsycINFO, ProQuest Central, and IEEE Xplore from database establishment up to May 29, 2026. The following search terms were utilized: (VR OR virtual* OR immersive*) AND (autis* OR ASD OR Asperger* OR pervasive developmental disorders) AND (RCT OR Random* OR Control* OR Blind* OR interventions).

### 2.3 Literature search

After completing comprehensive database retrieval, the authors recorded all retrieved citations and eliminated duplicate records. The initial screening relied on titles and abstracts, followed by full-text assessment against the pre-defined eligibility criteria. To ensure accuracy, two authors (Zhang & Luo) performed this procedure independently. Any disagreements were resolved through consensus discussion between the two independent raters. The inter-rater agreement between the two investigators reached 91%. The process and outcomes of the selection of studies are visually represented in the PRISMA flow diagram in Figure 1.

**Figure 1:**
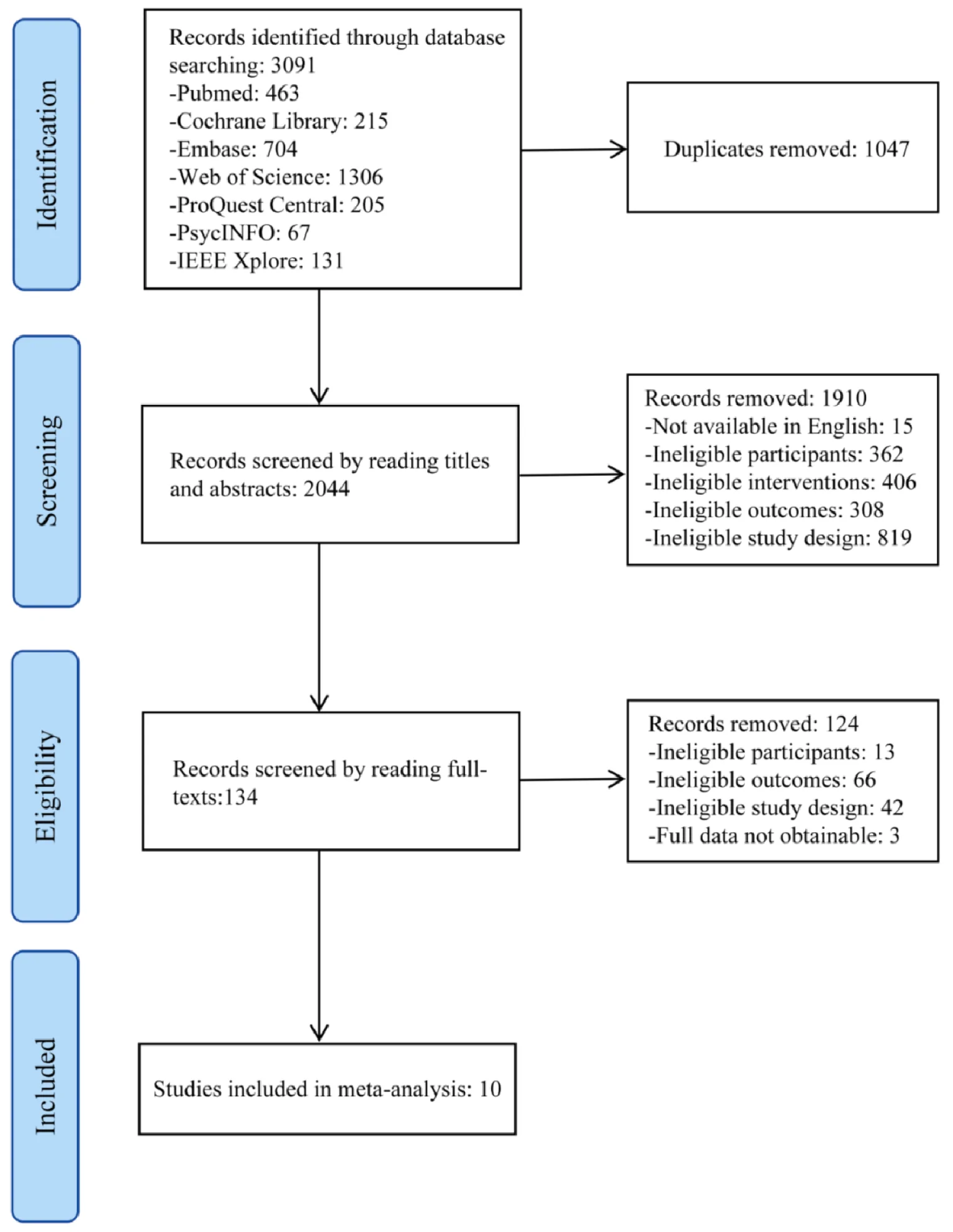
PRISMA flow diagram.

### 2.4 Quality assessment

Two authors (Zhang and Luo) independently assessed evidence quality and risk of bias. All unresolved discrepancies were judged by a third author (Chen). The inter-rater agreement coefficient between the two raters was 90%. The methodological quality of each outcome estimate was evaluated using the revised tool to assess risk of bias in randomized trials (RoB 2, Sterne et al., 2019). This tool evaluates the following five domains of potential bias: (1) Bias in the randomization process, (2) Bias owing to deviations from intended interventions, (3) Bias owing to missing outcome data, (4) Bias in measuring outcomes, and (5) Bias in selecting reported results. Each domain is assessed using specific signaling questions. If none of the items are likely to influence the results, the domain is scored as having a low risk of bias. If concerns exist regarding one or more items that may affect the validity of the results, the domain was rated as having some risk of bias (some concerns). If any item is judged to substantially impact the outcomes, the domain is scored as having a high risk of bias.

### 2.5 Data extraction

Well-being is a broad term encompassing optimal emotional, cognitive, social, and psychological functioning (Diener, 1984; Ryan and Deci, 2001). There are different ways to conceptualize well-being, such as Diener’s (1984) subjective well-being (SWB), Ryff’s (1989) psychological well-being (PWB), Keyes’s (1998) social well-being, and Seligman’s (2011) PERMA framework (which stands for Positive Emotion, Engagement, Relationships, Meaning, and Accomplishment). To date, Diener’s (1984) and Ryff’s (1989) frameworks have been more widely used in the positive psychology literature (Diener et al., 2018; Disabato et al., 2016; Joshanloo, 2019; Pang and Datu, 2025). Accordingly, these two models were adopted to categorize well-being outcomes in this meta-analysis. SWB involves people’s appraisals of their own life content, positive emotions, and negative emotions (Diener, 1984; Diener et al., 1999). PWB encompasses one’s perceived self-acceptance (acceptance of good and bad qualities of self), environmental mastery (competence in managing daily challenges), purpose in life (sense of purpose and meaningfulness in life), positive relations with others (warm, satisfying, and trusting interpersonal relationships), personal growth (sense of continued growth and improvement), and autonomy (sense of self-determination and personal authority) (Ryff, 1989; Ryff et al., 2021).

For the extraction of well-being-related outcomes, Pang and Datu (2025) offered several illustrative examples of such outcomes. For example, social interaction and anxiety (dental) were regarded as well-being-related outcomes in their research. In the present study, social interaction was specifically treated as an outcome linked to PWB, while anxiety (dental) was classified as an outcome related to SWB. While practical coding examples were drawn from Pang and Datu (2025), our categorization was primarily anchored to the core theoretical definitions proposed by Diener (1984) and Ryff (1989). Two authors (Zhang and Luo) independently conducted the extraction and classification of well-being-related outcomes. Any discrepancies were resolved by a third independent reviewer (Chen).

During data extraction, we first retrieved the mean values, standard deviations, and sample sizes of both the experimental and control groups at pre-test and post-test time points. Subsequently, the DECoMA tool—co-developed by Cochrane Taiwan and Wan Fang Hospital (available at https://taiwan.cochrane.org/resource/decoma)—was applied to calculate mean change scores (post-test scores minus pre-test scores) and their corresponding standard deviations. Meanwhile, this DECoMA tool was utilised to pool data from four subgroups reported in Frolli et al. (2022), namely Recognition of primary emotions, Recognition of secondary emotions, Emotions and situations for primary emotions, and Emotions and situations for secondary emotions, into a unified outcome measure of social skills. Two independent reviewers (Zhang and Luo) performed the data extraction process separately. Cross-verification of the extracted datasets demonstrated complete consistency between the two investigators.

In addition to extracting data for effect size calculation, we also collected basic characteristics of the included studies, including first authors, publication years, cultural contexts, baseline sample sizes, ages, genders, dropout rates, intervention contexts, intervention durations, well-being-related outcomes, their scoring methods (including forward scoring and reverse scoring), and the corresponding types of well-being (including SWB and PWB). This process was completed independently by two authors (Zhang and Luo). Any discrepancies were resolved through discussion to reach a consensus. The inter-rater agreement rate between the two raters was 96%.

### 2.6 Data analysis

All statistical analyses were performed using R software version 4.5.3. Meta-analyses were mainly performed using the meta, metafor, robvis, and ggplot2 packages.

The Hedges’g (Hedges and Olkin, 2014) was employed as the primary effect size. Given that this review incorporated numerous studies with small sample sizes (e.g., Genova et al., 2021; Ma and Song, 2025; Suresh and Shetty, 2026), this adjusted effect size is appropriate (Borenstein et al., 2021). The formula is as follows: g = (M_1_-M_2_)/SD_pooled_, where M_1_ denotes the mean change score of the intervention group, M_2_ represents the mean change score of the control group, and SD_pooled_ refers to the pooled and weighted standard deviation of change scores across the two groups (Hedges and Olkin, 2014). A positive Hedges’g value represents improved well-being. For scales where higher scores reflect poorer well-being, we reversed the sign of mean change scores during the calculation of effect sizes.

Given the differences in intervention durations, implementation settings, participant ages, and measurement instruments across studies included in this meta-analysis, clinical heterogeneity was observed among the eligible studies. The random-effects model accommodates such variation and allows generalizing conclusions to a wider population of relevant studies, rather than limiting inferences only to the included studies. Therefore, a random-effects model was applied separately to pool the overall effect sizes for outcomes related to SWB and PWB. Interpretation of effect sizes followed Cohen’s criteria (Cohen, 2013): values of 0.2, 0.5, and 0.8 represent small, moderate, and large effect sizes, respectively.

The heterogeneity among studies was evaluated using the Q test and *I*^2^ statistic. Cochran’s Q test (Cochran, 1954) was used to test statistical heterogeneity. Heterogeneity was considered statistically significant if *p* < 0.05. The *I*^2^ statistic was further applied to quantify the magnitude of heterogeneity. *I*^2^ < 25%, *I*^2^ of 25–50%, and *I*^2^ > 50% indicate low, moderate, and high heterogeneity, respectively (Higgins et al., 2003). High heterogeneity requires cautious interpretation of pooled effect estimates (West et al., 2010). In case of high heterogeneity between studies, subgroup analyses were performed to identify the variables contributing to the observed heterogeneity. It should be noted that the *I*^2^ reflects the statistical heterogeneity of effect-size estimates and does not rule out underlying clinical or conceptual heterogeneity.

Furthermore, sensitivity analyses were performed to evaluate the robustness of the pooled outcome and investigate the impact of individual studies on the overall effect magnitude. A leave-one-out sensitivity analysis was undertaken separately for outcomes related to SWB and PWB, whereby each effect size was sequentially excluded before the meta-analysis was reestimated. This method was employed to determine whether the overall pooled result was significantly affected by any single study and to verify the stability of our conclusion. If heterogeneity tests for the SWB and PWB subgroups indicate low statistical heterogeneity, a circumstance may emerge in which low statistical heterogeneity coexists with a certain degree of clinical heterogeneity. Should this scenario occur, a sensitivity analysis comparing fixed-effect and random-effect models would be performed. This procedure helps examine whether pooled effect estimates are sensitive to the selection of analytical models.

Finally, funnel plots and Egger’s tests (Egger et al., 1997) were separately employed for outcomes related to SWB and PWB to assess whether publication bias (i.e., the tendency for studies yielding statistically significant results to be more likely to be published than those reporting non-significant findings) might have influenced the results of this review. A symmetric funnel plot indicates a low degree of publication bias, whereas an asymmetric one suggests potential publication bias. The significance level for Egger’s test was set at 0.05. Egger’s test was adopted for the evaluation of publication bias. *P* < 0.05 was considered indicative of potential publication bias; *P* ≥ 0.05 indicated insufficient evidence to detect publication bias.

## 3 Results

The preliminary search identified 3,091 records: 463 from Pubmed, 215 from Cochrane Library, 704 from Embase, 1,306 from Web of Science, 205 from ProQuest Central, 67 from PsycINFO, and 131 from IEEE Xplore. After the removal of duplicates (1,047 records) and excluding studies through reading their titles and abstracts (1,910 records), and through assessing full texts (124 records), the search procedure retrieved 10 eligible studies (Figure 1).

### 3.1 Characteristics of included studies

Table 1 summarizes the key characteristics of the 10 eligible studies, including research designs, participants, interventions, and well-being-related outcomes.

**Table 1:** Characteristics of included studies (*n* = 10).

Study	VR hardware types	VR immersion levels	Cultural contexts	Baseline_N	Age span	Gender	Drop-out	intervention contexts	durations (weeks)	Outcomes		
										Scoring methods	Detailed outcome	Types of well-being
Al Kheraif et al. (2024)	HMDs	Immersive	KSA	IG: 70; CG: 70	4–18	M: 105; F: 35	IG: 0; CG: 0	Clinical	<1	RS	Anxiety (dental)	SWB
Chu et al. (2023)	DVRE	Non-immersive	China	IG: 39; CG: 39	3–6	M: 61; F: 17	IG: 0; CG: 0	Clinical	20	RS	Social skills (social relating)	PWB
Frolli et al. (2022)	DVRE	Non-immersive	Italy	IG: 30; CG: 30	9–10	M: 51; F: 9	IG: 0; CG: 0	School	12	FS	Social skills	PWB
Genova et al. (2021)	DVRE	Non-immersive	USA	IG: 7; CG: 7	16–19	M: 13; F: 1	IG: 0; CG: 0	School	8	FS	self-efficacy (job interview)	PWB
Ip et al. (2018)	CAVE	Immersive	Hong Kong	IG: 36; CG: 36	6–12	M: 64; F: 8	IG: 0;CG: 0	School	14	FS FS	Emotion regulation Social interaction	PWB PWB
Ip et al. (2024)	HMDs	Immersive	Hong Kong	IG: 48; CG: 59	6–12	M: 88; F: 19	IG: 0; CG: 0	School	15	FS	Social interaction	PWB
Ma and Song (2025)	HMDs	Immersive	China	IG: 10; CG: 10	8–13	M: 11; F: 9	IG: 1; CG: 0	Clinical	16	RS RS	Depression Anxiety	SWB SWB
Maskey et al. (2019)	CAVE	Immersive	UK	IG: 16; CG: 16	8–14	M: 25; F: 7	IG: 0; CG: 1	Clinical	24	RS RS	Fear Anxiety	SWB SWB
Sepehri Bonab et al. (2025)	DVRE	Non-immersive	Iran	IG: 20; CG: 20	7–10	M: 40; F: 0	IG: 0; CG: 0	Clinical	8	FS RS	Emotion regulation Negative emotion	PWB SWB
Zhao et al. (2022)	HMDs	Immersive	China	IG: 22; CG: 22	3–5	M: 35; F: 9	IG: 0; CG: 0	Clinical	12	FS	Social interaction	PWB

IG = intervention group; CG = control group; M = male; F = female; FS = forward scoring; RS = reverse scoring. The articles were listed alphabetically by the surname of the first author.

Incorporated in the present meta-analysis were ten empirical studies published from 2018 to 2025. These investigations covered a broad spectrum of cultural backgrounds, including the Kingdom of Saudi Arabia, mainland China, Hong Kong, Italy, the United States, the United Kingdom, and Iran, which strengthen the cross-cultural generalizability of the pooled effect sizes derived from the integrated quantitative synthesis.

All research samples targeted children aged 3 to 19 years. Male participants constituted the majority across most trials, revealing notable gender imbalance within the included samples; specifically, one study conducted in Iran recruited solely male participants with no female participants involved. The aggregated baseline samples across all eligible studies comprised 607 participants, including 493 males and 114 females, with males making up 81% of the overall samples.

Of the ten included studies, participant attrition was observed in merely two trials. Ma and Song (2025) documented one dropout within the intervention group, while Maskey et al. (2019) recorded one dropout in the control group. No participants withdrew from the remaining eight studies over the entire course of intervention implementation.

Interventions were implemented in two distinct types of settings: clinical environments and school campuses. Six of the ten included studies administered interventions in clinical contexts, whereas the other four adopted school-based intervention protocols. The shortest intervention duration was reported in the study by Al Kheraif et al. (2024), which consisted of a single 10-min session. By contrast, the longest intervention lasted 24 weeks in the research conducted by Maskey et al. (2019).

Among the ten included studies, a total of 14 well-being-related outcome indicators were extracted. Specifically, six outcomes corresponded to SWB, and eight pertained to PWB. These 14 well-being outcomes were evenly split between forward scoring and reverse scoring methods. For all reverse-scored indicators, higher scores reflected lower levels of well-being. Accordingly, the signs of mean change scores for both the intervention and control groups were inverted during the calculation of effect sizes for these seven reverse-scored outcomes.

### 3.2 Risk of bias assessment

Figure 2 presents the evaluation of methodological quality of the included studies and their corresponding potential biases. For the methodological characteristics of the 10 eligible RCTs in the present meta-analysis, 2 studies (20%) received an overall low risk-of-bias rating, 7 studies (70%) were categorised as having some concerns, and the remaining 1 study (10%) was assessed to carry a high overall risk of bias. Overall methodological quality across the 10 eligible RCTs was moderate. Very few eligible studies achieved an overall low risk-of-bias classification, with the bulk of trials categorised as having some concerns.

**Figure 2:**
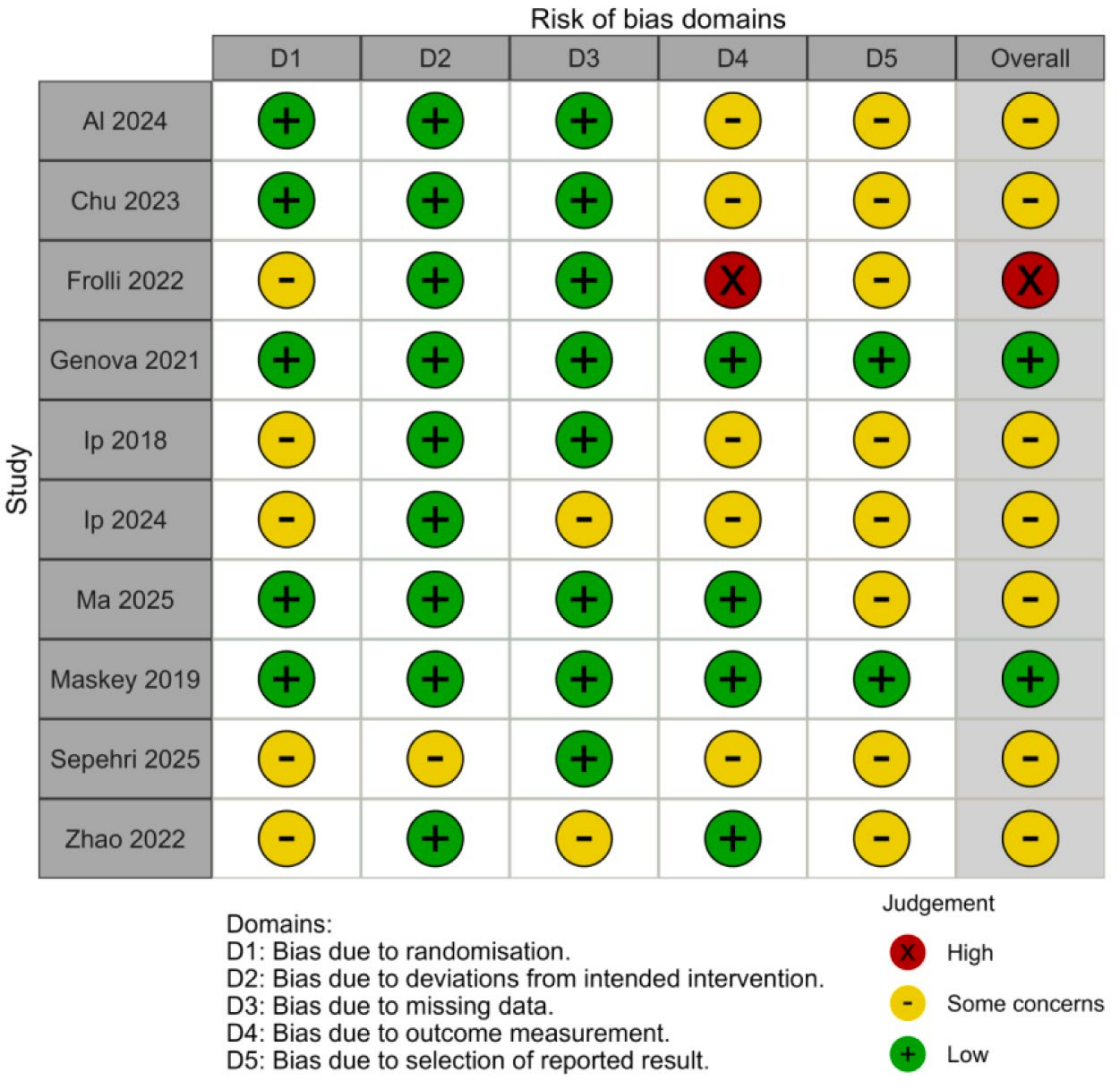
Risk of bias summary plot.

### 3.3 Overall effect size

The aim of this study was to meta-analyse the effects of VR interventions on SWB-related outcomes and PWB-related outcomes in autistic children. Therefore, effect sizes were pooled separately for the SWB and PWB subgroups. Figure 3 (1) and (2) show the forest plots of the meta-analysis examining the effects of VR interventions on SWB-related outcomes and PWB-related outcomes among autistic children, respectively. In the meta-analytic forest plot, Ma 2025 (a) and Ma 2025 (b) refer to the SWB-related depression and anxiety outcomes in Ma and Song (2025), Maskey 2019 (a), and Maskey 2019 (b) indicate the SWB-related fear and anxiety outcomes in Maskey et al. (2019), Ip 2018 (a), and Ip 2018 (b) stand for the PWB-related emotion regulation and social interaction outcomes in Ip et al. (2018). These notations carry the same meaning in the leave-one-out sensitivity analysis forest plot.

**Figure 3:**
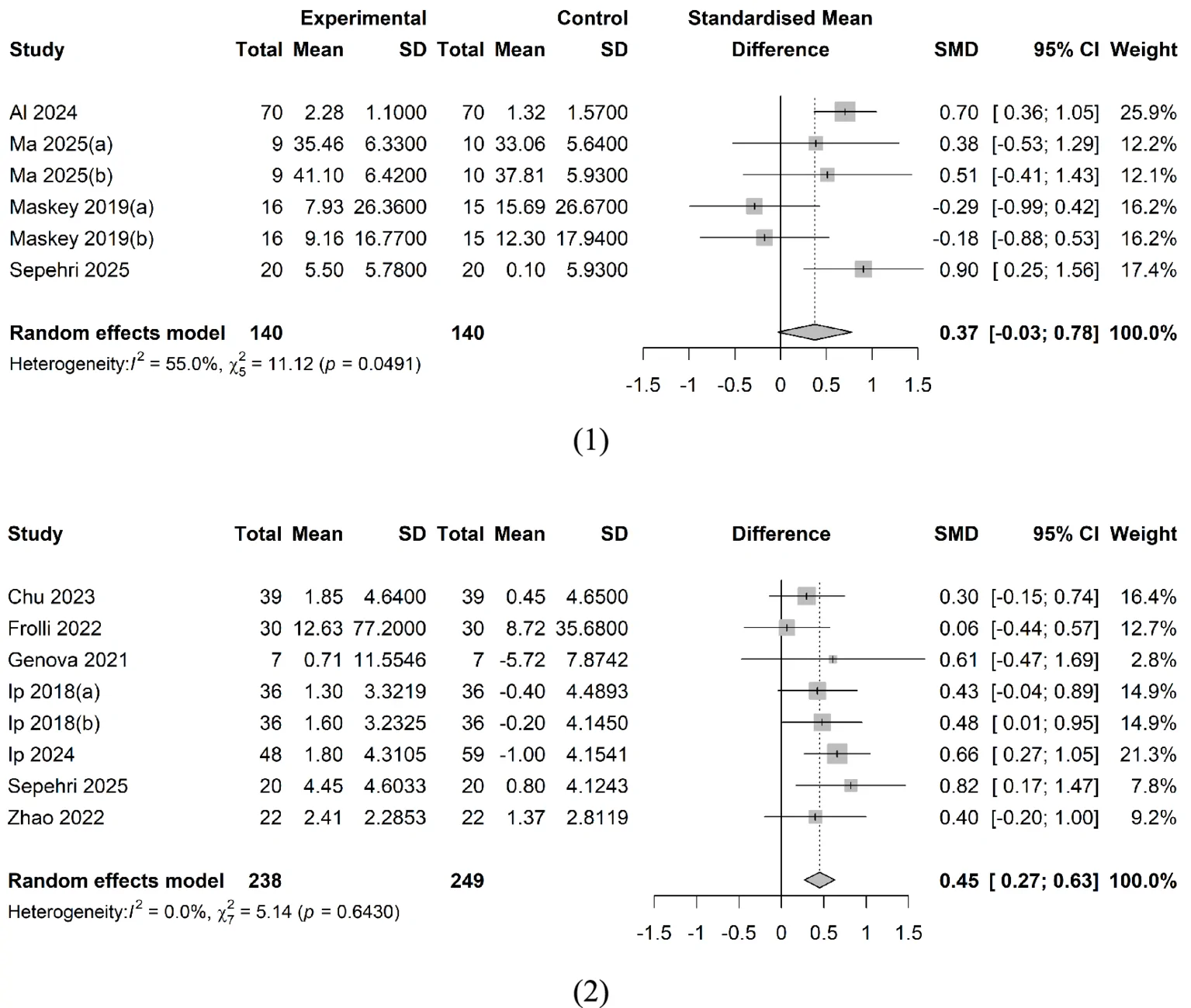
Meta-analysis forest plot for (1) SWB-related outcomes and (2) PWB-related outcomes.

The meta-analytic results revealed that virtual reality interventions yielded a small to moderate pooled effect on SWB (g = 0.37, 95% CI [−0.03, 0.78], z = 1.80, *p* = 0.07 > 0.05) and a statistically significant small to moderate pooled effect on PWB (g = 0.45, 95% CI [0.27, 0.63], z = 4.87, *p* < 0.05). The aggregated effect for SWB crossed the null value, indicating nonsignificant overall intervention effects, whereas the pooled estimate for PWB demonstrated a meaningful beneficial effect, with its 95% confidence interval entirely situated above zero. Individual primary studies exhibited substantial variability in point estimates for SWB, ranging from g = −0.29 to g = 0.90, while effect sizes for PWB were uniformly positive across included studies and varied within a narrower range (g = 0.06 ~ 0.82).

Noticeable between-study heterogeneity was observed for SWB-related outcomes (*I*^2^ = 55%, Q = 11.12, df = 5, *p* = 0.0491 < 0.05), indicating high heterogeneity and justifying the use of the random-effects model. Conversely, no statistically significant heterogeneity was detected for PWB-related outcomes (*I*^2^ = 0%, Q = 5.14, df = 7, *p* = 0.6430). Notably, an *I*^2^ value of 0% indicates low statistical heterogeneity among the included studies; nevertheless, clinical heterogeneity still exists, which stems from variations in intervention durations, VR hardware types, VR immersion levels, and participant ages.

### 3.4 Sensitivity analysis

To evaluate the influence of individual studies on the overall effect size, leave-one-out sensitivity analyses were performed for both the SWB subgroup and PWB subgroup. The analytical results are presented in Figure 4.

**Figure 4:**
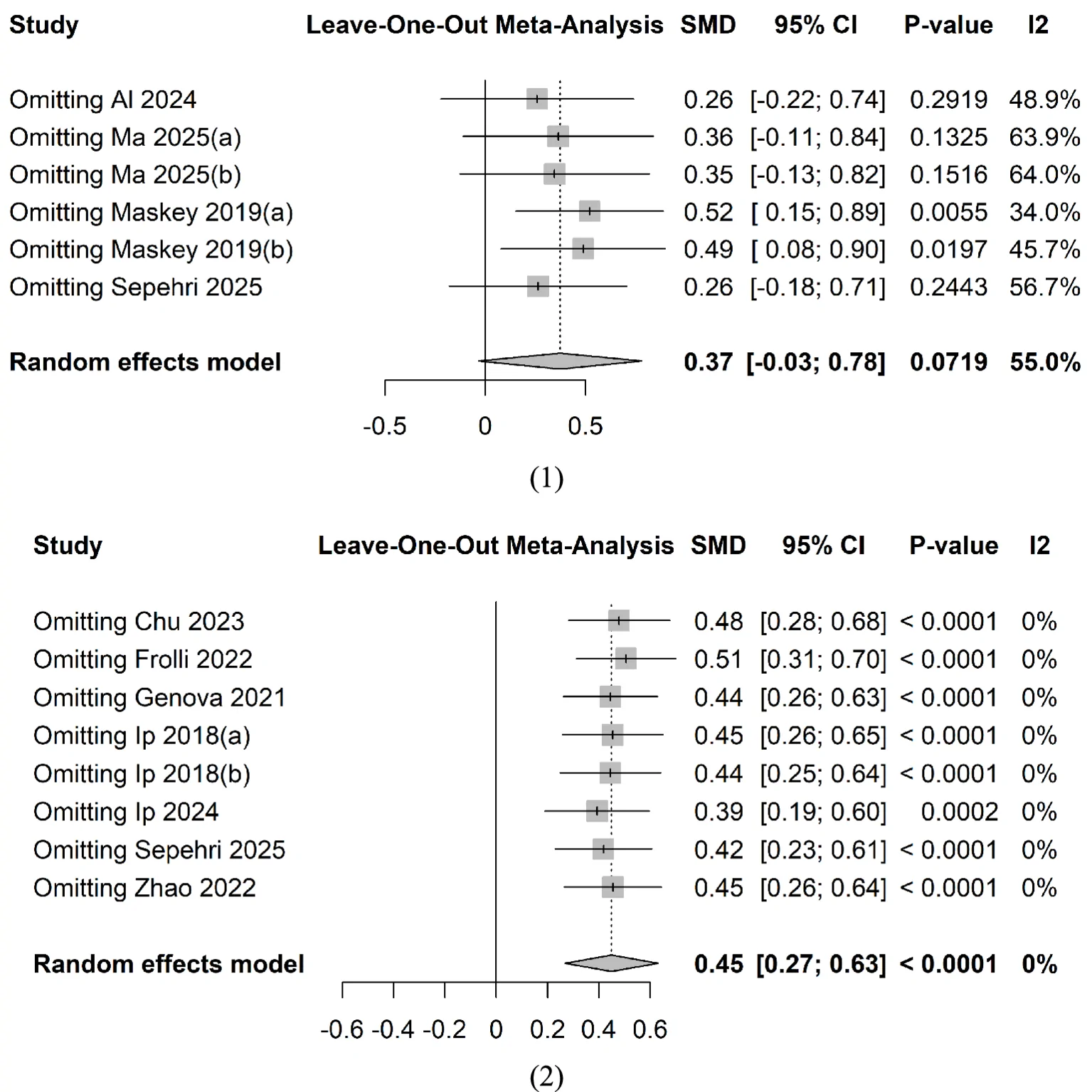
Leave-one-out sensitivity analysis forest plot for (1) SWB-related outcomes and (2) PWB-related outcomes.

Figure 4 (1) displays results for the SWB subgroup. Omitting individual studies sequentially yielded g values ranging from 0.26 to 0.52. When removing Maskey (2019a), the pooled effect increased to g = 0.52, 95% CI [0.15, 0.89], *p* = 0.0055, with between-study heterogeneity reduced to *I*^2^ = 34.0%. Excluding Maskey (2019b) also produced a statistically significant pooled estimate (g = 0.49, 95% CI [0.08, 0.90], *p* = 0.0197, *I*^2^ = 45.7%). By contrast, omission of Al Kheraif et al. (2024) or Sepehri Bonab et al. (2025) attenuated the overall effect size to g = 0.26 and yielded nonsignificant *p*-values. Across all leave-one-out iterations, 95% confidence intervals consistently crossed zero in most scenarios. Heterogeneity fluctuated between 34.0 and 64.0% after single study deletion. Collectively, these findings suggested that the pooled effect for SWB was methodologically fragile: statistical significance was conditional on the inclusion or exclusion of specific primary studies, and substantial unexplained between-study heterogeneity persisted after leave-one-out filtering.

Figure 4 (2) shows the analytical results for the PWB subgroup. Leave-one-out sensitivity analyses generated pooled Hedges’g values spanning 0.39 to 0.51. All recalculated 95% confidence intervals remained entirely above zero, and all corresponding p-values reached statistical significance (p < 0.05). Between-study heterogeneity stayed at I2 = 0% for every iteration. Elimination of any single study did not materially shift the direction, magnitude, or statistical significance of the summary effect. Accordingly, the effect of VR interventions on PWB-related outcomes was not sensitive to the exclusion of any single study.

As indicated by the preceding heterogeneity test, no substantial heterogeneity was detected within the subgroup of PWB-related outcomes (*I*^2^ = 0%, Q = 5.14, df = 7, *p* = 0.6430). Low statistical heterogeneity was detected within this subgroup, notwithstanding the presence of clinical heterogeneity. Accordingly, a sensitivity analysis comparing fixed-effect and random-effect models was conducted to examine whether the statistically significant positive effect of VR interventions on PWB-related outcomes among autistic children was driven by model specification. Under the fixed-effect model, the pooled effect size for PWB-related outcomes was g = 0.45 (95% CI [0.27, 0.63], z = 4.87, *p* < 0.05), demonstrating that the significant beneficial effect of VR interventions on PWB was not sensitive to model specification.

In the sensitivity analyses for PWB-related outcomes, both leave-one-out sensitivity analysis and comparative sensitivity analysis between fixed-effect and random-effects models indicated that the analytical results for PWB-related outcomes were robust. Nevertheless, given that the overall methodological quality of the 10 eligible RCTs was moderate and the number of included effect sizes was limited (6 for SWB and 8 for PWB), the robustness of the study conclusions should not be overestimated.

### 3.5 Publication bias

Potential publication bias was evaluated through funnel plot and Egger’s test. Figure 5 (1) and (2) display the funnel plots for publication bias for the SWB subgroup and PWB subgroup, respectively. Egger’s regression test did not reach statistical significance for either SWB (t = −1.17, df = 4, *p* = 0.31 > 0.05) or PWB (t = 0.24, df = 6, *p* = 0.82 > 0.05). Nevertheless, Egger’s test is known to possess low statistical power when fewer than 10 effect sizes are available per subgroup. Consequently, the nonsignificant Egger’s test result cannot reliably rule out publication bias. Funnel plots for both subgroups exhibited mild asymmetry, which may signal small-study effects or latent publication bias. Taken together, we cannot rule out potential publication bias in this meta-analysis, given limited power of Egger’s test with few effect sizes and mild funnel-plot asymmetry.

**Figure 5:**
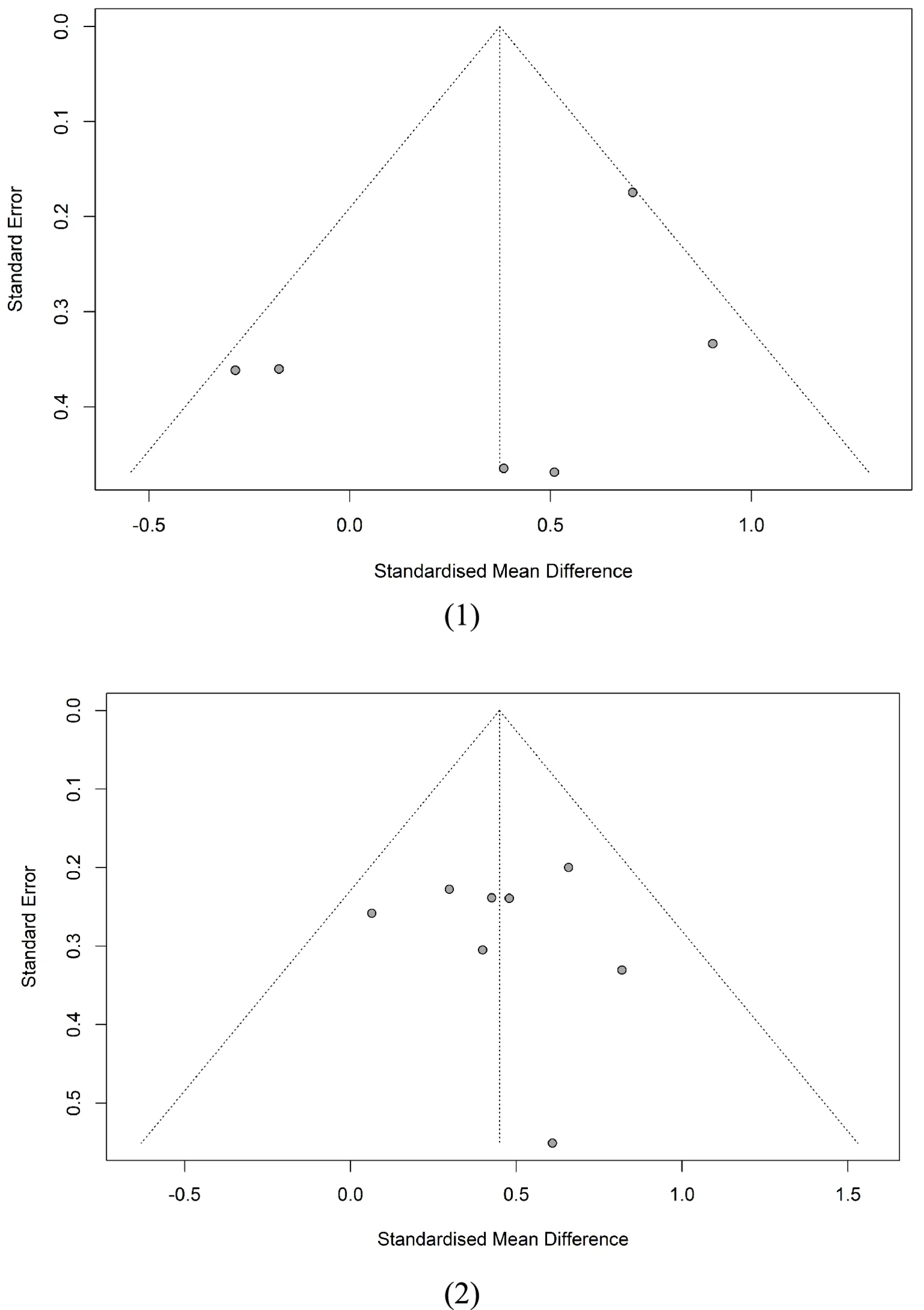
Funnel plot for publication bias for (1) SWB-related outcomes and (2) PWB-related outcomes.

## 4 Discussion

Previous meta-analyses have examined the effects of VR interventions on skills (Karami et al., 2021; Guan et al., 2026; Sun et al., 2026; Wang et al., 2026) and emotions among autistic children (Mesa-Gresa et al., 2018; Herrero and Lorenzo, 2020; Li et al., 2023; Farashi et al., 2024; Mittal et al., 2024; Yang et al., 2025; Cui et al., 2026). Based on data from 10 RCTs, this meta-analysis examined the effects of VR interventions on SWB-related outcomes and PWB-related outcomes among autistic children. We synthesized evidence from these 10 RCTs and calculated the pooled effect sizes of VR interventions separately. The main findings of the present study are discussed below.

First, meta-analytic pooling found no statistically significant VR-driven improvements in SWB outcomes (g = 0.37, 95% CI [−0.03, 0.78], z = 1.80, *p* = 0.07 > 0.05). This non-significant pooled estimate stemmed from substantial cross-study variability in effect sizes, which ranged widely from −0.29 to 0.90. Sensitivity analysis indicated that the statistical significance of this finding was highly dependent on specific studies, suggesting fragile results. This was primarily attributable to two SWB-related outcomes (fear and anxiety) extracted from Maskey et al. (2019), both of which produced negative effect sizes [see Maskey 2019 (a) and Maskey 2019 (b) in Figure 3 (1)] thereby elevating heterogeneity among included studies within SWB subgroup. In the study by Maskey et al. (2019), the intervention effect sizes of VR for the outcomes of fear and anxiety were negative, which may be attributable to the questionnaires utilised. The authors noted that the suitability of these questionnaires as outcome measures requires further examination (Maskey et al., 2019).

Omitting these two outcomes from Maskey et al. (2019) and repooling effect sizes for the SWB subgroup revealed a statistically significant beneficial effect of VR (g = 0.69, 95% CI [0.42, 0.97], z = 4.95, *p* < 0.05). Accordingly, the ameliorative effect of VR on SWB-related outcomes warrants further examination when more relevant empirical evidence becomes available in future research.

Furthermore, this meta-analysis found an interesting phenomenon within the subgroup of SWB-related outcomes. Discrepant intervention effects of VR were observed for anxiety outcomes in autistic children. Al Kheraif et al. (2024) and Ma and Song (2025) reported positive VR effect sizes for anxiety reduction [g = 0.70 and g = 0.51, respectively; Figure 3 (1)], demonstrating VR’s capacity to lower anxiety symptoms. In contrast, Maskey et al. (2019) yielded a negative effect size (g = −0.18) for anxiety outcomes, meaning the VR protocol in their trial failed to reduce anxiety severity. Further research is warranted to explore VR intervention effects on anxiety among autistic children and disentangle the underlying drivers of these inconsistent findings.

In addition, the present meta-analysis indicated that VR interventions exerted a significant positive effect on PWB-related outcomes. According to Cohen’s guidelines (Cohen, 2013), the pooled effect size of 0.45 (95% CI [0.27, 0.63], z = 4.87, *p* < 0.05) is considered small to moderate. The overall effect test indicated that the pooled effect reached statistical significance (*p* < 0.05). The heterogeneity statistic (Q = 5.14, df = 7, *p* = 0.64; *I*^2^ = 0%) revealed low heterogeneity across the included studies. Leave-one-out sensitivity analyses demonstrated that no single study unduly affected the pooled effect size. A sensitivity analysis comparing fixed-effect and random-effect models also indicated that the analytical results were nearly insensitive to model specification. The test for publication bias indicated that Egger’s test was not statistically significant (t = 0.24, df = 6, *p* = 0.82 > 0.05). Notwithstanding the low statistical heterogeneity and satisfactory sensitivity test results for this pooled estimate, several important constraints must be underscored: 70% of the included RCTs were rated as having some concerns for risk of bias, and only eight effect sizes were synthesized for PWB outcomes. These limitations preclude strong claims of clinical readiness. Therefore, although VR shows preliminary promise for improving PWB among autistic children, its real world clinical utility still requires further validation from larger, methodologically rigorous trials.

Although we observed an *I*^2^ = 0%, indicating an absence of statistically detectable between-study variation in pooled effect sizes, this statistical finding does not equate to clinical homogeneity across the included studies. In fact, clinical heterogeneity objectively exists given the discrepancies among the included studies in terms of intervention duration, VR immersion levels, and age range. For this reason, a random-effect model was adopted for pooling effect sizes in the present meta-analysis.

The pooled outcome constructs within the PWB subgroup, including self-efficacy and social interaction, appear to represent disparate outcome measures. Nevertheless, all combined outcome constructs fall within the core dimensions of Ryff’s theoretical framework of PWB. For instance, self-efficacy corresponds to the dimensions of autonomy and environmental mastery, whereas social interaction reflects positive relations with others. While these outcomes exhibit differential measurement performance, they all operationalize facets of the overarching latent construct of PWB. Accordingly, pooling these indicators to generate a primary aggregated effect size is theoretically justified for addressing our core research question: What is the overall effect of VR-based interventions on PWB-related facets in autistic children?

Finally, a marked gender imbalance was noted among pooled baseline participants from the 10 included studies, with male participants constituting 81% and female participants only 19% of the total sample. This finding is consistent with prior research, which has repeatedly demonstrated a higher prevalence of ASD in males relative to females (Beggiato et al., 2017; Ferri et al., 2018; Wang et al., 2022; Farashi et al., 2024), with the latest reported male-to-female ratio reaching 3:1 (Coury et al., 2020). This further validates the viewpoint proposed by Werling and Geschwind (2013) that ASD exhibits a pronounced male bias.

This meta-analysis has a number of limitations. First, this meta-analysis is limited by the small sample of eligible RCTs (*n* = 10), yielding 14 unique well-being-related effect sizes (6 for SWB, 8 for PWB). The limited number of effect sizes prohibited moderator analyses for key variables such as participant ages, VR hardware types, VR immersion levels, intervention durations, and comorbid mental health conditions. Included studies exhibited heterogeneity with respect to the variables of VR immersion levels. The significant PWB effect and the fragile SWB effect of VR may be disproportionately driven by immersive versus non-immersive interventions. Divergent VR immersion levels may produce differing intervention effects on PWB and SWB. Nonetheless, subgroup analyses for VR immersion levels were not feasible, because the small number of included studies would yield underpowered and unstable estimates that preclude valid statistical inference. Accordingly, we could not determine whether immersive or non-immersive VR accounted for the observed effects, leaving this heterogeneity associated with VR immersion levels untested. Future meta-analyses drawing on larger RCT samples are required to examine whether VR immersion levels moderate VR-induced improvements in well-being among autistic children.

Another limitation is that we excluded studies without complete extractable data for effect size calculation. The non-inclusion of such studies may influence the intervention effect of VR on the well-being-related outcomes of autistic children. In future research, it is crucial to incorporate studies with limited data that enable effect size calculations—by contacting the authors of previously published research—to provide a more comprehensive exploration of the impact of VR on the well-being-related outcomes of autistic children.

Additionally, assessment of publication bias constitutes another limitation of this synthesis. Each subgroup only contained a limited number of effect sizes (6 for SWB, 8 for PWB). Under such conditions, Egger’s test has limited statistical power to detect true publication bias even if it exists. Although Egger’s test yielded non-significant *p*-values, we observed mild asymmetry in both funnel plots. This asymmetry may stem from publication bias, small-study effects, or other sources of between-study heterogeneity. We therefore cannot draw firm conclusions regarding the absence of publication bias. This potential bias should be kept in mind when interpreting the pooled effect-size estimates for SWB and PWB.

Lastly, this meta-analysis was further limited by the fact that most included studies exhibited partial concerns for risk of bias upon RoB 2 evaluation (Higgins et al., 2024; Sterne et al., 2019). Specifically, although only 1 study (10%) was classified as high-risk, only 2 studies (20%) were deemed low-risk, while the remaining 7 studies (70%) presented certain concerns. Overall, the methodological rigor of the included studies was restricted: few trials were devoid of methodological flaws, and the majority demonstrated varying degrees of bias that could potentially impact the reliability of the pooled results. These methodological characteristics highlight the necessity for more rigorous methodological designs in future VR intervention studies.

## 5 Conclusion

Previous meta-analyses have synthesized effect sizes of VR interventions on well-being-related outcomes among autistic children, including emotional recognition (Cui et al., 2026), emotional skills (Mittal et al., 2024), cognitive skills (Mittal et al., 2024), and social skills (Mittal et al., 2024; Yang et al., 2025; Cui et al., 2026). The meta-analysis conducted by Pang and Datu (2025) addressed the research question “Can digital assistive technology interventions foster well-being among students with disabilities?” By contrast, the present study focuses on the intervention effects of VR on outcome measures associated with SWB and PWB for autistic children. Based on moderate quality evidence from randomized controlled trials, VR interventions demonstrated a small to moderate statistically significant pooled effect for PWB-related outcomes. This finding warrants cautious interpretation given that most trials had some concerns in risk of bias assessment and only eight effect sizes were available for synthesis. The evidence supports preliminary research-level promise for improving PWB, rather than immediate clinical readiness for routine practice. For SWB, by contrast, no conclusive evidence was found to support VR interventions, and further large scale RCTs are required to validate the potential SWB benefits of VR.

## Data Availability

The original contributions presented in the study are included in the article/supplementary material, further inquiries can be directed to the corresponding author/s.
